# High-sensitivity Gd^3+^–Gd^3+^ EPR distance measurements that eliminate artefacts seen at short distances

**DOI:** 10.5194/mr-1-301-2020

**Published:** 2020-12-09

**Authors:** Hassane EL Mkami, Robert I. Hunter, Paul A. S. Cruickshank, Michael J. Taylor, Janet E. Lovett, Akiva Feintuch, Mian Qi, Adelheid Godt, Graham M. Smith

**Affiliations:** 1 SUPA, School of Physics and Astronomy, University of St Andrews, St Andrews, KY16 9SS, UK; 2 Department of Chemical Physics, Weizmann Institute of Science, Rehovot, Israel; 3 Faculty of Chemistry and Center of Molecular Materials (CM_2_), Bielefeld University, Universitätsstraße 25, 33615 Bielefeld, Germany

## Abstract

Gadolinium complexes are attracting increasing attention as spin labels for EPR dipolar distance measurements in biomolecules and particularly for in-cell measurements. It has been shown that flip-flop transitions within the central transition of the high-spin Gd ${}^{3+}$ ion can introduce artefacts in dipolar distance measurements, particularly when measuring distances less than 3 nm. Previous work has shown some reduction of these artefacts through increasing the frequency separation between the two frequencies required for the double electron–electron resonance (DEER) experiment. Here we use a high-power (1 kW), wideband, non-resonant system operating at 94 GHz to evaluate DEER measurement protocols using two stiff Gd(III) rulers, consisting of two bis -Gd ${}^{3+}$ –PyMTA complexes, with separations of 2.1 nm and 6.0 nm, respectively. We show that by avoiding the $\left|-\frac{1}{2}\rangle \to \left|\frac{1}{2}\rangle \right.\right.$ central transition completely, and placing both the pump and the observer pulses on either side of the central transition, we can now observe apparently artefact-free spectra and narrow distance distributions, even for a Gd–Gd distance of 2.1 nm. Importantly we still maintain excellent signal-to-noise ratio and relatively high modulation depths. These results have implications for in-cell EPR measurements at naturally occurring biomolecule concentrations.

## 1 Introduction

Double electron–electron resonance (DEER) spectroscopy combined with site-directed spin labelling (SDSL) is a powerful technique to probe structural and dynamic properties in a wide range of biological systems. Over the past decades, distance measurements have been mainly associated with nitroxide spin labels (SLs). This has led to the development of new experimental protocols and reliable data analysis programs for a routine extraction of distances and investigation of conformational changes. Amongst other reasons, the increasing interest in characterising proteins in their native environment has extended the spin labelling family to new labels based on paramagnetic metal ion complexes. Gd ${}^{3+}$ -based SLs have been of particular interest as they already exist as a major class of contrast agents used in MRI and show a strong stability against the oxidation or reduction conditions found in cells, making them an ideal candidate for in-cell distance measurements.

Gadolinium is a half integer high-spin S=7/2 metal ion, characterised by a broad distribution of zero-field-splitting (ZFS) parameters and an isotropic g value at high field (Raitsimring et al., 2005). At lower temperatures its EPR spectrum is dominated by the central transition $\left|-\frac{1}{2}\rangle \to \left|\frac{1}{2}\rangle \right.\right.$ superimposed on a broad featureless background coming from all the other transitions. To first order, perturbation theory shows the central transition is independent of the ZFS interaction, while the other transitions scale linearly with the axial ZFS parameter D . However, to second order the central line narrows as the operational frequency increases and its width scales proportionally with $\frac{D^{2}}{gB_{0}}$ . Therefore, high frequencies have been favoured for distance measurements using Gd ${}^{3+}$ -based spin labels due to an expected improved concentration sensitivity associated with placing the pump or observer frequency at the central line.

Since their introduction in 2007, several Gd ${}^{3+}$ -based spin labels have been developed and a wide range of molecules have been successfully investigated, from simple model compounds to proteins, DNA, peptides and other biological systems (Gordon-Grossman et al., 2011; Potapov et al., 2010; Raitsimring et al., 2007; Yagi et al., 2011; Shah et al., 2019; Wojciechowski et al., 2015). The good agreement between distance distributions derived from Gd–Gd DEER data and those resulting from other techniques has motivated researchers to attempt investigation of in-cell proteins and peptides (Qi et al., 2014; Yang et al., 2019; Dalaloyan et al., 2019; Martorana et al., 2014). In most of these studies, Gd ${}^{3+}$ was treated like an S=1/2 system and standard data analysis software packages, developed initially for nitroxides, have generally been applied. This approach has proven successful for Gd–Gd distances above 3–4 nm, but below 3–4 nm strongly damped dipolar distortions and artificially broadened distance distributions were obtained (Cohen et al., 2016; Dalaloyan et al., 2015; Feintuch et al., 2015; Manukovsky et al., 2017). This is caused by unwanted flip-flop transitions, whose effects are enhanced by strong dipolar coupling (Cohen et al., 2016; Manukovsky et al., 2017). This effect can be ameliorated by increasing the frequency offset between the pump and observer pulses (PO offset) (Cohen et al., 2016). This has usually been achieved by having the pump pulse positioned at the central transition and positioning the observer pulse with as large a frequency offset as possible. This is usually difficult to achieve with standard resonator bandwidths on commercial instruments. Nevertheless, a high-frequency dual-mode cavity with an ingenious design has been demonstrated, which can accommodate pump and observer pulses with separations of more than 1 GHz (Cohen et al., 2016). Unfortunately such cavities, particularly at high fields and low temperatures, can be challenging to set up precisely. Relaxation-induced dipolar modulation enhancement (RIDME) is another experimental alternative where no such restrictions apply, since it is a single-frequency technique. However, it suffers from overtones of dipolar frequencies and requires a more complicated data analysis (Collauto et al., 2016; Keller et al., 2017; Meyer and Schiemann, 2016; Razzaghi et al., 2014).

In the present work, we demonstrate a simpler approach that uses a wideband non-resonant or weakly resonant sample holder to show the benefit of wideband measurements. We use two Gd rulers with calculated distances between two Gd(III)–PyMTA complexes of 2.1 and 6.0 nm (Qi et al., 2016; Dalaloyan et al., 2015). The Gd–Gd distances were calculated for a temperature at 160.4 K, the glass transition temperature of the mixture of glycerol-d ${}_{8}$ and D ${}_{2}$ O, 50/50 ( v/v ), applying the wormlike chain model as described previously (Dalaloyan et al., 2015). We explore two different experimental protocols. The standard approach is where the pump pulse is positioned at the central transition, but with variable offset between pump and observer pulses of up to 900 MHz. In general, we observe narrower distance distributions and improved Pake patterns as frequency separation is increased. In the second approach we place the pump and observer pulses on either side of the $\left|-\frac{1}{2}\rangle \to \left|\frac{1}{2}\rangle \right.\right.$ central transition, avoiding excitation of the central transition altogether. In this case, we observe almost perfect Pake patterns, consistent with elimination of the artificial broadening of the distance distribution, even for the 2.1 nm Gd ruler.

For this short 2.1 nm distance we show that any loss of sensitivity from not exciting the central transition is offset by the shorter time window now required to make the measurement.

## 2 Experiment

### 2.1 Sample preparation

**Figure 1:**
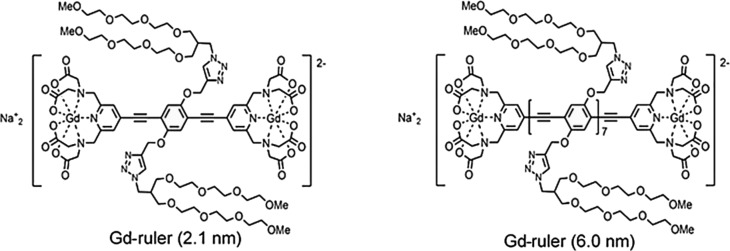
Chemical structures of Gd-ruler systems used in the current study.

The synthesis of the Gd rulers has been described elsewhere (Qi et al., 2016). Solutions of 40 µM concentration (molecules) of the Gd ruler (2.1 nm) and Gd ruler (6.0 nm) were prepared in 50/50 ( v/v ) deuterated glycerol and D ${}_{2}$ O (for chemical structure see Fig. 1). The use of the glycerol-d ${}_{8}/$ D ${}_{2}$ O ( 1:1 , v/v ) was dictated by the desire to obtain a good glass, to reduce scattering losses and to extend the phase memory time. For the Q-band measurements the samples were transferred to standard 3 mm quartz EPR tubes and flash-frozen in liquid nitrogen prior to loading into the spectrometer. For the W-band experiments, the samples were transferred into 27 mm long fluorinated ethylene propylene (FEP) tubes with 3 mm outer diameter and 2 mm inner diameter and flash-frozen in liquid nitrogen prior loading into sample-holder cartridges that were separately pre-cooled in liquid nitrogen. These sample-holder cartridges were then loaded into the W-band spectrometer which had been pre-cooled to 150 K.

### 2.2 Spectrometers

The spectrometers used for these measurements were a Bruker ELEXSYS E580 high-power (150 W) Q-band pulsed spectrometer with ER 5106QT-2W resonator and a home-built 1 kW W-band spectrometer (93.5–94.5 GHz). This W-band spectrometer, widely known as HiPER, has been described previously (Cruickshank et al., 2009; Motion et al., 2017). It operates with a wideband non-resonant (or weakly-resonant) induction mode sample holder, which is now described in more detail.

#### 2.2.1 Non-resonant induction mode sample holder

A non-resonant induction mode sample holder is essentially a shorted symmetrical waveguide where two dominant orthogonal linearly polarised modes can propagate. The incident linearly polarised beam can be decomposed into two orthogonal circular polarisation components. At resonance, one circular component is absorbed (or emitted) by the sample, resulting in a reflected beam containing a cross-polarised component perpendicular to the linear polarisation of the incident beam. In the system described here these reflected signals are diplexed via a wire grid polariser to separate the high-power incident beam from the very much smaller cross-polarised component which is passed to the detection system. The dimensions of the empty waveguide sample holder (3 mm diameter) are chosen to be single-mode at the operating frequency of the spectrometer. The advantage of these shorted waveguide sample holders is that they are inherently wideband since they are only weakly resonant. It might be thought that sensitivity would be strongly diminished as there is no resonant enhancement of either the excitation pulse or signal. However, the critical parameter that determines the resonant enhancement is the microwave conversion efficiency of the sample holder, c , with units of gauss per root watt (Smith et al., 2008). A single-mode shorted waveguide at 94 GHz can have a comparable or better conversion efficiency (typically about 0.5 G/W ${}^{1/2}$ at the sample) than a commercial X-band pulsed resonator. Compared to a dual-mode W-band resonator, a shorted waveguide can also offer a hugely increased sample volume (up to 75 µL in the current system), provided that sample dielectric losses are low, which is usually the case for measurements made at cryogenic temperatures. The non-resonant cavity also offers considerable flexibility in optimising excitation frequencies and bandwidths at both pump and observer frequencies. Therefore these types of systems can have extremely high concentration sensitivity whilst offering very large instantaneous bandwidths. This potentially makes them ideal for Gd SL distance measurements, especially when large separations between the observer and pump pulses are required.

The sample is placed in a FEP tube within a sample-holder cartridge and mounted into a spring-loaded mount (see Fig. S1a in the Supplement). The latter co-locates to a smooth cylindrical waveguide transmission line of diameter 3 mm, which supports two orthogonal TE ${}_{11}$ modes. Radiation is fed to the shorted waveguide via an adapted corrugated feed horn that feeds to a wider bore corrugated pipe which in turn feeds to an optical system. An adjustable backshort, consisting of a roof mirror with a shallow roof angle, is placed in the waveguide below the sample. Adjustment of this backshort allows optimisation of the cross-polarised signal component, which is important both for initial experimental set-up and for measurements. This adjustment is achieved using piezo motors (Attocube Systems AG) that separately control the roof angle and the relative distance of the backshort to the sample.

To facilitate the loading of pre-frozen samples, the samples and sample-holder cartridges are pre-cooled externally to the spectrometer in liquid nitrogen. The spring-loaded mount, feed horn and corrugated pipe are housed inside a vacuum vessel which forms an extension to the sample flow cryostat and includes a vacuum window at the top to allow access for the microwave beams. The sample cryostat is cooled until the temperature of the spring-loaded mount reaches 150 K, which has been found to be a reliable temperature to use for loading of pre-frozen samples. In order to load the sample, the flow cryostat must be stopped and returned to ambient pressure using helium gas. The vacuum vessel is hoisted up along with the corrugated pipe, feed horn and spring-loaded mount whilst a continuous flow of helium gas is maintained to minimise icing of the cryostat and microwave feed system. The sample-holder cartridge is removed from the liquid nitrogen and inserted into the spring-loaded mount along a guide channel where it becomes located into sockets, ensuring accurate alignment of the waveguide. The vacuum vessel is then lowered back down and sealed to the cryostat and is then evacuated. Cryogen flow is reinstated in the cryostat and the system is cooled to the measurement temperature.

#### 2.2.2 Frequency-dependent power variation in HiPER

It should be noted that the transmitted power level (from the amplifier–isolator–switch combination) is not constant over the whole range of the frequency offsets used in this study. This is illustrated in Fig. S1b where we show the power level versus frequency monitored at different points along the transmitter chain of HiPER. As a consequence of this, absolute modulation depths should not be compared quantitatively.

### 2.3 Pulse EPR experiments

For the Q-band experiments, echo detected field sweep (ED-FS) measurements were carried out at 10 K. The π/2 and π pulse lengths were set at 16 and 32 ns respectively, with an inter-pulse delay of τ=200 ns.

For the W-band experiments all measurements were performed at 10 K, which corresponds to the optimum temperature for these experiments when the central transition is excited (Feintuch et al., 2015; Goldfarb, 2014; Raitsimring et al., 2013). The ED-FS spectra were recorded using a Hahn echo sequence with pulse lengths 6 and 12 ns as π/2 and π respectively and a delay of 300 ns. These pulse lengths were optimised by setting the magnetic field to the peak of the spectrum. The echo decay ( $T_{m}$ ) and the inversion recovery ( $T_{l}$ ) experiments were recorded at the central maximum of the ED-FS spectrum by stepping the associated sequences with steps of 100 ns and 1 µs respectively. It should be noted that differences are expected particularly for the phase relaxation when measuring away from the central transition (Raitsimring et al., 2014). The repetition rate for all W-band experiments was set at 3 kHz, unless otherwise stated, and this was again optimised at the maximum of the ED-FS spectrum.

The DEER experiments were carried out using the standard dead-time free four-pulse sequence (Pannier et al., 2000).

$$\begin{array}{rl} & \frac{\pi }{2}\left(\text{obs}\right)\to \tau_{1}\to \pi \left(\text{obs}\right)\to t\to \pi \left(\text{pump}\right)\to \tau_{1}+\tau_{2}-t\\ & \to \pi (\text{obs})\to \tau_{2}\to \text{echo} \end{array}$$

The echo intensity was monitored as a function of t . For the Gd ruler (6.0 nm), the pump pulse was applied, for all experiments, at the maximum of the ED-FS spectrum, whereas the observer frequency was set at 94 GHz with different offsets from the pump frequency as reported in Table 1. For the Gd ruler (2.1 nm), different settings were investigated, with either the pump frequency being set at the maximum of the ED-FS spectrum and the observer frequency placed on the side of the central line or with both the pump and observer frequencies being set on either side of the central line. The experimental parameters used in both cases are summarised in Table 1. Optimisation of the observer and pump pulse lengths was carried out systematically for each experiment given the wide range of frequency offsets used in this study. It is necessary to re-optimise the pulse lengths for each frequency offset, due to power variation from the transmitter chain. For technical reasons, these measurements were made without phase cycling, but instead offsets were removed by separate automatic measurements of the baseline on either side of the echo (at a slight cost in SNR).

**Table 1:** Settings for W-band DEER measurements on both rulers and the associated modulation depths obtained by fitting the DEER data with DeerAnalysis (2019) (Jeschke et al., 2006). The interpulse delay $\tau_{1}$ was set to 300 ns for all experiments. To allow different DEER measurements to be compared more easily we take our sensitivity measure as the echo SNR multiplied by the modulation depth divided by the square root of the total number of measurements. It should be noted that this does not take into account differences in excitation bandwidth of pump and observer pulses.

	Offset ${}^{a}$	Obs ${}^{b}$	Pump ${}^{c}$	$\tau_{2}$	Data	SRR ${}^{d}$	λ	Echo	Time	Number of	Sensitivity
	(MHz)	π (ns)	π (ns)	( µs )	points	(kHz)	(%)	SNR	averaging	averages ${}^{e}$	measure ${}^{f}$
Pumping on the central line and observing on the side											
Gd ruler	120 (P ${}_{1}$ O ${}_{1}$ )	11	10	10.3	251	3	6.0	1111	1 h 30 min (14 scans)	42 000	2.06
(6.0 nm)	120 (P ${}_{2}$ O ${}_{2}$ )	16	16	10.3	251	3	5.0	769	44 min (7 scans)	21 000	1.68
	420 (P ${}_{3}$ O ${}_{3}$ )	11	10	10.3	251	3	4.5	2000	10 h 20 min (99 scans)	297 000	1.02
Gd ruler	120 (P ${}_{1}$ O ${}_{1}$ )	24	24	2.2	251	3	2.5	1923	1 h (10 scans)	30 000	1.75
(2.1 nm)	420 (P ${}_{2}$ O ${}_{2}$ )	12	12	2.8	251	3	4.0	3125	1 h (10 scans)	30 000	4.56
	840 (PO ${}_{5}$ )	12	12	1.5	121	1	2.9	3279	1 h 40 min (33 scans)	33 000	4.68
	900 (PO ${}_{6}$ )	12	12	1.5	121	1	3.2	3846	0 h 45 min (15 scans)	15 000	8.99
Pumping and observing on the sides of the central line											
Gd ruler	800 (P ${}_{3}$ O)	8	8	1.5	121	1	2.1	8333	1 h 26 min (28 scans)	28 000	9.35
(2.1 nm)	900 (P ${}_{4}$ O)	12	12	1.5	121	1	1.1	10 000	4 h 27 min (71 scans)	71 000	3.69

${}^{a}1$ Frequency separation between pump pulse set at position i (P ${}_{i}$ ) and observer pulse at position j (O ${}_{j}$ ). ${}^{b,\;c}$ Observer and pump π pulse lengths. The observer π/2 pulse was always half the observer π pulse. ${}^{d}$ SRR is shot repetition rate. ${}^{e}$ Number of averages calculated as number of scans × number of shots per point. ${}^{f}$ The sensitivity measure is calculated as $=\frac{\lambda \cdot \text{SNR}(\text{Echo})}{\left(\sqrt{\text{total number of points measured}}\right)}$ , where SNR (echo) is the ratio of the maximum echo height to the standard deviation of the noise. This is obtained by subtracting a smoothed fit from the data and then calculating the standard deviation from the resulting noise trace. The total number of points measured is the total number of averages per point multiplied by the number of points in a scan.

DEER data were processed using the DeerAnalysis (2019) program that allows extraction of distance distributions (Jeschke et al., 2006). Fits to the data were based on standard Tikhonov regularisation analysis using the bending point in the L-curve. The excitation profiles of the pump and observer pulses were simulated using a home-written MATLAB-based program using simple spin mechanics (Jeschke and Polyhach, 2007). The simulated ED-FS spectra and the associated sub-spectra for each transition were performed using the EasySpin program (Stoll and Schweiger, 2006).

## 3 Results

### 3.1 EPR spectra and relaxation times

**Figure 2:**
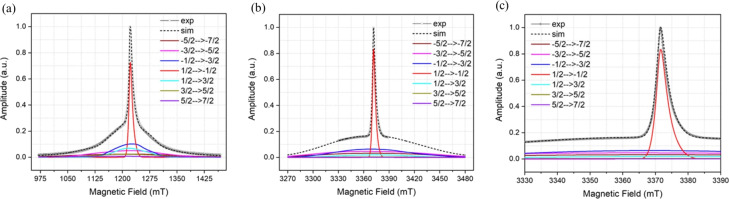
Simulated and experimental ED-FS spectra of the Gd ruler (6.0 nm) with the associated sub-spectra of the individual transitions, **(a)** at Q-band, **(b)** at W-band with wide magnetic field ranges and **(c)** at W-band with narrow magnetic field range respectively. The parameters used in the simulation are D=1060 MHz, $D_{\mathrm{strain}}=850$ MHz, E=320 MHz and $E_{\mathrm{strain}}=200$ MHz.

The ED-FS spectra for both samples are similar to those reported for other Gd ${}^{3+}$ complexes with a characteristic sharp line corresponding to the central transition and a broad featureless background resulting from contributions of all other transitions. The spectra recorded at Q- and W-bands are shown in Fig. 2. The simulation of the sub-spectra was performed using EasySpin by considering a distribution of the ZFS parameters D and E (Stoll and Schweiger, 2006). The magnitude and distribution of the ZFS depend primarily on the nature of the interactions between the Gd ${}^{3+}$ ion and the ligand and/or solvent molecules coordinating to the Gd ${}^{3+}$ ion. These are taken into account by the D and E strain parameters used by EasySpin, and they are defined as monomodal Gaussian distributions. Furthermore, it was shown in some cases that a bimodal Gaussian distribution centred on D and -D considerably improved the simulation (Raitsimring et al., 2005; Clayton et al., 2018). The D parameters used for the simulations are reported with those obtained for other Gd ${}^{3+}$ tags in Table S1 in the Supplement.

The phase memory time, $T_{m}$ , and the spin lattice relaxation time, $T_{1}$ , were measured for both samples at 10 K with the magnetic field set on the central maximum of the ED-FS, and results were similar to other reported studies on Gd ${}^{3+}$ complexes measured at low temperatures (Collauto et al., 2016; Raitsimring et al., 2014). The $T_{m}$ time traces were fitted initially with a sum of two exponential functions with free exponent values, and excellent fits were achieved with fixed exponent values of 1 and 2, and the results are shown in Fig. S2a. The fit to two exponentials ( $R^{2}=0.9999$ ) was rather better than could be achieved by fitting to a stretched exponential. $T_{m}$ values estimated from these fits are shown in Table S2 and provide evidence for fast and slow relaxation contributions to the echo decay. It is emphasised that these relaxation data were taken at the central maximum, but $T_{m}$ relaxation is expected to be faster away from the central maximum due to transition-dependent fluctuations in the zero field splitting (Raitsimring et al., 2014).

$T_{1}$ time traces were also well fitted to a bi-exponential function as shown in Fig. S2b. Fast and slow time constants were derived from these fits and are reported in Table S3.

### 3.2 Results from DEER spectroscopy

#### 3.2.1 Gd ruler (6.0 nm)

**Figure 3:**
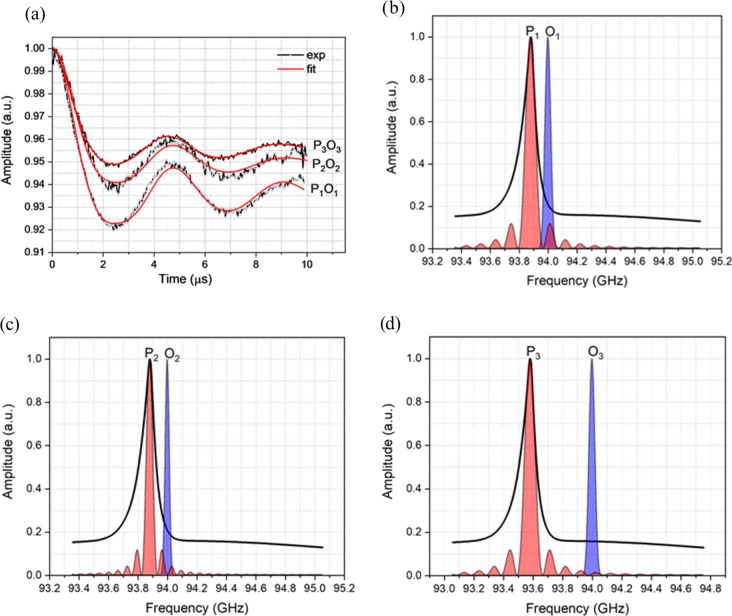
**(a)** Background-corrected DEER data (black curves) of Gd ruler (6.0 nm) recorded with different PO offsets with the fits (red) obtained by DeerAnalysis (2019) (Jeschke et al., 2006). Excitation profiles of the pump (P) and observer (O) at different frequency offsets, **(b, c)** P ${}_{1}$ O ${}_{1}=$ P ${}_{2}$ O ${}_{2}=120$ MHz with softer pulses for P ${}_{2}$ O ${}_{2}$ and **(d)** P ${}_{3}$ O ${}_{3}=420$ MHz.

Background-corrected DEER data obtained with Gd ruler (6.0 nm) are shown in Fig. 3a, and the corresponding primary data are shown in Fig. S3a. The pump and observer positions with their associated excitation profiles are reported in Fig. 3b, c and d. In addition to the excitation profiles, the pump and observer pulse positions, with respect to the central transitions $\left|-\frac{1}{2}\rangle \to \left|\frac{1}{2}\rangle \right.\right.$ , are shown in Fig. S4. The modulation depths derived from the fits are summarised in Table 1. The data and fits at low-frequency offsets are very similar to those measured before at W-band, allowing for differences in SNR and modulation depth (Dalaloyan et al., 2015). The modulation depth λ of 6 % obtained from the DEER data recorded with 120 MHz PO offset (see Table 1) is also in a good agreement with that derived from the concentration dependence of a similar parent Gd ${}^{3+}$ tag (Dalaloyan et al., 2015). By being slightly more selective with the pump pulse but keeping the same PO offset of 120 MHz, the modulation depth decreases to 5 % as expected due to fewer spins being excited. When the PO offset is increased to 420 MHz the modulation depth drops to 4.5 % mainly due to the output power drop off towards the band edges in our system. For this latter measurement, it should be noted the field was different than in the former two experiments, and the pump pulse is a different frequency (see Fig. 3b, c and d).

**Figure 4:**
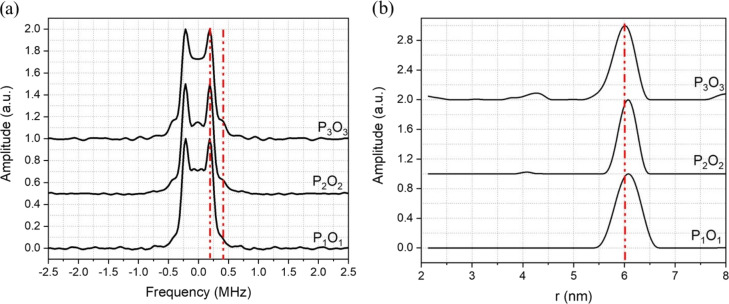
**(a)** Pake pattern spectra obtained from fitting of the DEER data of the Gd-ruler (6.0 nm) sample measured with different offsets between pump and observer pulses and **(b)** corresponding distance distributions derived from the DEER data. The PO offsets are P ${}_{1}$ O ${}_{1}=$ P ${}_{2}$ O ${}_{2}=120$ MHz and P ${}_{3}$ O ${}_{3}=420$ MHz. The red vertical dashed lines show in **(a)** the positions of the parallel and perpendicular singularities of the Pake pattern and in **(b)** the expected distance.

The derived Pake pattern spectra and the associated distance distributions are shown in Fig. 4. The distance distribution derived from DEER data measured with 120 MHz PO offset appears to be deviating slightly from 6.0 nm, the expected distance for this Gd ruler, with a full width at half height (FWHH) of 0.56 nm, whereas with 420 MHz offset it is well centred on 6.0 nm with a FWHH of 0.48 nm. The Pake patterns, for all experimental settings, show normal and typical shapes with clear dipolar singularities corresponding to parallel and perpendicular orientations. These DEER measurements were recorded, as mentioned, with the pump position set at the peak of the FS-ED spectrum, which primarily excites the $\left|-\frac{1}{2}\rangle \to \left|\frac{1}{2}\rangle \right.\right.$ transition, whereas the observer frequency for both offsets was positioned where the $\left|-\frac{3}{2}\rangle \to \left|-\frac{1}{2}\rangle \right.\right.$ transition contributes most to the detected signal (see Fig. S4a, b). The Gd ${}^{3+}$ spectrum is the result of a superposition of several transitions with different weights, and their contributions, either to the pumped and observed spins, are expected to be magnetic field dependent. By increasing the PO offset, while keeping the pump position at the maximum of the FS-ED spectrum, the contribution of the $\left|-\frac{3}{2}\rangle \to \left|-\frac{1}{2}\rangle \right.\right.$ transition to the detected signal gradually decreases whilst the contributions of the other transitions, $\left|-\frac{7}{2}\rangle \to \left|-\frac{5}{2}\rangle \right.\right.$ , $\left|-\frac{5}{2}\rangle \to \left|-\frac{3}{2}\rangle \right.\right.$ , increase.

#### 3.2.2 Gd ruler (2.1 nm)

**Figure 5:**
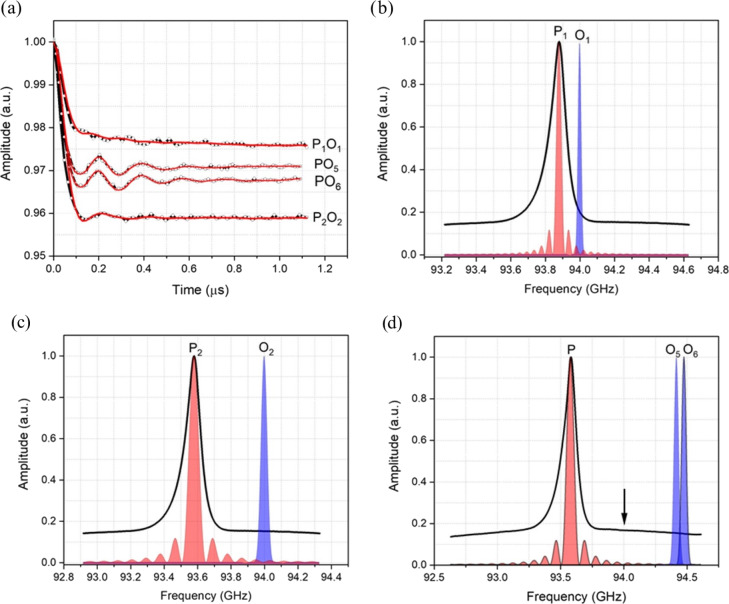
**(a)** Background-corrected DEER data (black curves) of Gd ruler (2.1 nm) recorded with different offsets between pump and observer pulses together with fits (red curves) obtained by DeerAnalysis (2019) (Jeschke et al., 2006). **(b, c, d)** Excitation profiles of the pump (P) and observer (O) pulses at different frequency offsets. Please note the different frequency scales. The corresponding frequency offsets are P ${}_{1}$ O ${}_{1}=120$ MHz, P ${}_{2}$ O ${}_{2}=420$ MHz, PO ${}_{5}=840$ MHz and PO ${}_{6}=900$ MHz. The black arrow indicates the position of 94 GHz, the nominal centre frequency of our W-band EIK amplifier, which has a bandwidth of just less than 1 GHz.

The DEER measurements with Gd ruler (2.1 nm) were conducted with a combination of different pump and observer positions and several PO offsets. Figure 5a shows background-corrected DEER data obtained with measurements performed with the pump pulse set at the position of the central transition with PO offsets of 120, 420, 840 and 900 MHz. The corresponding primary DEER data are shown in Fig. S3b. The excitation profiles of the pump and the observer at these positions are reported in Fig. 5b, c and d. The pump and observer positions with respect to the central transition are shown in Fig. S5. At 120 and 420 MHz PO offsets, the time domain DEER data show severely damped dipolar modulations (see Fig. 5a), whereas in the cases of 840 and 900 MHz offsets, the dipolar modulations are significantly recovered. However, they are still not as well defined as one might expect for a stiff model system. The obtained modulation depths λ are reported in Table 1.

**Figure 6:**
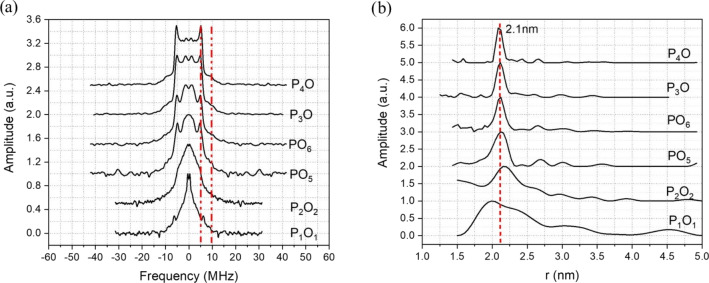
**(a)** Pake pattern spectra obtained from the fitting of the DEER data of the Gd-ruler (2.1 nm) sample measured with different offsets between pump and observer pulses and **(b)** associated distance distributions derived from the DEER data. The corresponding frequency offsets are P ${}_{1}$ O ${}_{1}=120$ MHz, P ${}_{2}$ O ${}_{2}=420$ MHz, PO ${}_{5}=840$ MHz, PO ${}_{6}=900$ MHz, P ${}_{3}$ O =800 MHz and P ${}_{4}$ O =900 MHz. The red vertical dashed lines show in **(a)** the positions of the parallel and perpendicular singularities of the Pake pattern and in **(b)** the expected distance.

The Pake pattern spectra reported in Fig. 6a show strong distortions and poorly resolved dipolar singularity points for the 120 and 420 MHz PO offsets. In contrast we observe substantially improved Pake pattern spectra for the larger offsets of 840 and 900 MHz, particularly concerning the perpendicular dipolar singularities. In Fig. 6b, the distance distributions are considerably broadened for the 120 and 420 MHz PO offsets, with FWHH, determined only for the major peak centred at 2.1 nm, of 0.83 and 0.45 nm. However, at 840 MHz PO offset, the peak in the distance distribution is centred at the expected 2.1 nm distance with a FWHH of 0.24 nm. The best results were obtained with the 900 MHz PO offset giving a distance distribution with a FWHH of only 0.17 nm. Note that results for 840 and 900 MHz are given as this is close to the edge of the extended interaction klystron (EIK) amplifier bandwidth.

**Figure 7:**
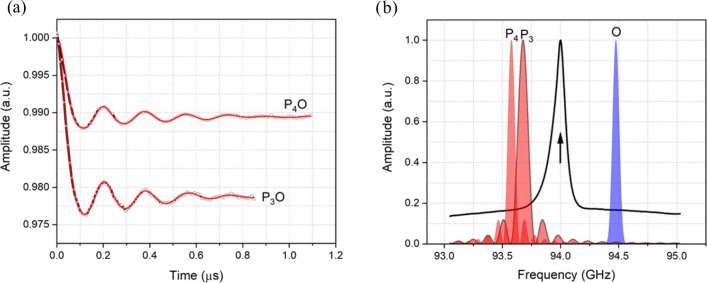
**(a)** Background-corrected DEER data (black curves) of Gd ruler (2.1 nm) recorded with different offsets between pump and observer pulses together with fits (red curves) obtained by DeerAnalysis (2019) (Jeschke et al., 2006). **(b)** Excitation profiles of the pump (P) and observer (O) pulses at different frequency offsets. The corresponding frequency offsets are P ${}_{3}$ O =800 MHz and P ${}_{4}$ O =900 MHz. The black arrow indicates the position of 94 GHz, the nominal centre frequency of our W-band EIK amplifier, which has a bandwidth of just less than 1 GHz.

A further set of DEER experiments were performed by setting the pump and observer pulses on either side of the central transition with large PO offsets. With this we aimed to exclude completely the contribution of the $\left|-\frac{1}{2}\rangle \to \left|\frac{1}{2}\rangle \right.\right.$ transition of both the pumped and observed spins (see Fig. S6). Figure 7a shows the DEER data corresponding to 800 and 900 MHz frequency PO offsets. The pulse profiles associated with the pump and observer pulses are presented in Fig. 7b. For both PO offsets the obtained dipolar modulations show more than four clear oscillations and smooth damping, highly reminiscent of spectra of structurally related nitroxide rulers with similar distances (Godt et al., 2006).

The Pake patterns reported in Fig. 6a show the expected shape with well-resolved perpendicular and parallel dipolar singularities. The corresponding distance distributions, shown in Fig. 6b, show a very narrow major peak centred at 2.1 nm with FWHH of 0.17 and 0.11 nm respectively. We also checked for signs of asymmetry in the distance distribution associated with flexibility of the ruler backbone, as previously found with nitroxide rulers (Godt et al., 2006). This asymmetry is not clearly evident with Gd ruler (2.1 nm), possibly because the ruler is too short, as mentioned by one of the referees. However, there are signs of asymmetry for Gd ruler (6.0 nm) in the measurement corresponding to P ${}_{3}$ O ${}_{3}$ at large pump observer offsets. This asymmetry becomes less clear at smaller pump observer offsets as can be seen from Fig. 4. This is further evidence that even at long distances it can be beneficial to have large offsets between pump and observer.

## 4 Discussion

High-quality DEER spectra from 40 µM concentration samples were obtained with averaging times of an hour or two. Modulation depth, SNR of the echo and experimental parameters are given in Table 1. As different traces were measured with a different number of scans and different shot repetition times, or have different number of points in the scan, we also provide a sensitivity measure for DEER measurements that normalises for these quantities. Results can be compared to W-band measurements on the same Gd rulers (Dalaloyan et al., 2015). The high concentration sensitivity, relative to conventional W-band resonator-based spectrometers, is attributed to much larger effective sample volumes ( ∼50 –80 µL ) and larger excitation bandwidths facilitated by the high available power that is only partially offset by the lower conversion factor. Large effective volumes are possible with biological systems in non-resonant sample holders at W-band, as dielectric losses are expected to be small at cryogenic temperatures ( tan⁡δ<0.001 ) if a high-quality glass is formed.

The echo decays (measured at the central transition), shown in Fig. S2a, were well fitted with a sum of two exponential functions with exponents found to be extremely close to 1 and 2 ( $R^{2}=0.9999$ ). Little difference in phase memory time was observed between the two rulers, with a slightly lower decay for Gd ruler (2.1 nm). However, no correlation had been found between the echo decay rate and the Gd–Gd distance of the same type of ruler as used in our study (Dalaloyan et al., 2015). This type of decay appears to be a characteristic of the Gd ${}^{3+}$ complexes, and very similar results have been obtained before (Collauto et al., 2016; Raitsimring et al., 2014; Dalaloyan et al., 2015). Nuclear spin diffusion is often the dominant process in phase relaxation of the central transition (Garbuio et al., 2015), when one would expect the data to be well fitted with a single stretched exponential with an exponent of close to 2 (Kathirvelu et al., 2009). However nuclear spin diffusion is expected to be significantly reduced by matrix deuteration, and contributions resulting from thermally assisted fluctuations in the zero-field splitting are then expected to become significant (Raitsimring et al., 2014). The need to fit with two stretched exponentials suggests an additional fast dephasing process is contributing to the transverse relaxation. We tentatively speculate that this additional dephasing process is driven by intra-molecular instantaneous diffusion due to the electron spin flip-flop processes resulting from simultaneous excitation of $\left|-\frac{1}{2}\rangle \to \left|\frac{1}{2}\rangle \right.\right.$ transitions belonging to both Gd ${}^{3+}$ ions of one Gd ruler. This seems to be consistent with the $T_{m}$ values derived from the fits (see Table S2) which show identical slow parts for both samples as one would expect for the same matrix and different fast parts as a result of two Gd ${}^{3+}$ systems with different dipolar couplings, due to different Gd–Gd distances, and therefore different spin flip-flop rates. The inter-molecular instantaneous diffusion process is less effective at concentrations as low as those used in this study and is therefore considered to not contribute. However, this hypothesis needs further investigation at different concentrations and on a similar compound with a single Gd ${}^{3+}$ label.

Inversion recovery data shown in Fig. S2b have been well fitted with the sum of two mono-exponential functions with fast and slow components (see Table S3). In addition, we provided a single $T_{1}$ value that was determined from a mono-exponential function fit to the inversion recovery data. It is interesting to note that the longer $T_{m}$ component is comparable to the shorter $T_{1}$ component.

For DEER experiments, the weak coupling approximation is generally expected to hold when the PO offset between the coupled spins is significantly larger than the dipolar coupling between the coupled spins. This is usually fulfilled for Gd ${}^{3+}$ –Gd ${}^{3+}$ distance measurements where a frequency offset of at least 100 MHz is often used. In both Gd rulers, Gd ruler (2.1 nm) and Gd ruler (6.0 nm), the expected dipolar couplings are 5.6 and 0.2 MHz respectively and are far below the smallest frequency offset of 120 MHz used in our measurements. However, in the present work, as well as in the literature, artefacts in the spectra are observed for distances below 3–4 nm (Raitsimring et al., 2007; Dalaloyan et al., 2015; Cohen et al., 2016; Manukovsky et al., 2017). Such artefacts mainly manifest themselves as a damping of the dipolar modulations in the time domain, which in turn results in an artificial broadening of the distance distributions. This has previously been explained in terms of unwanted excitation of flip-flop transitions within the central line. For the highest sensitivity in Gd ${}^{3+}$ –Gd ${}^{3+}$ DEER measurements, the pump pulse is usually set at the maximum of the ED-FS spectrum to ensure the deepest modulation depth (and the observer is often set just outside the central line). Under such conditions, the central transition, $\left|-\frac{1}{2}\rangle \to \left|\frac{1}{2}\rangle \right.\right.$ , contributes most to the pumped spins, whereas, just away from the central transition, the $\left|-\frac{3}{2}\rangle \to \left|-\frac{1}{2}\rangle \right.\right.$ transition becomes the more dominant contribution to the observer spins (see Fig. S4a, b). The DEER signal is thus the result of the difference between the energy levels associated with the two transitions $\left|-\frac{3}{2}\left(A\right),\frac{1}{2}(B)\rangle \to \left|-\frac{1}{2}\left(A\right),\frac{1}{2}(B)\rangle \right.\right.$ and $\left|-\frac{3}{2}\left(A\right),-\frac{1}{2}(B)\rangle \to \left|-\frac{1}{2}\left(A\right),-\frac{1}{2}(B)\rangle \right.\right.$ . The associated energies of these two states are degenerate to first order of the ZFS, and only a fairly small splitting is induced by the second-order ZFS contribution; this falls within the range of the dipolar couplings corresponding to short distances between Gd ${}^{3+}$ ions. Therefore, the weak coupling approximation is no longer satisfied and the secular pseudo-terms describing the flip-flop effects cannot be ignored. This has been confirmed theoretically and investigation has shown that the artefacts are only significant for short distances, where the dipolar coupling is large, and when either the pump or observer pulse is set on the $\left|-\frac{3}{2}\rangle \to \left|-\frac{1}{2}\rangle \right.\right.$ transition adjacent to the $\left|-\frac{1}{2}\rangle \to \left|\frac{1}{2}\rangle \right.\right.$ or vice versa (Cohen et al., 2016; Manukovsky et al., 2017). However, when other transitions are selected by the observer pulse, it was shown that these artefacts are strongly reduced (Manukovsky et al., 2017). This was originally experimentally confirmed in experiments with a dual-mode cavity (Cohen et al., 2016) and is also clearly seen in the experiments described here. This is particularly demonstrated in Fig. 5 where the pump pulse is set on the $\left|-\frac{1}{2}\rangle \to \left|\frac{1}{2}\rangle \right.\right.$ transition and the observer pulse is moved progressively further away from the central transition, which gradually reduces the contribution of the adjacent $\left|-\frac{3}{2}\rangle \to \left|-\frac{1}{2}\rangle \right.\right.$ transition (see Fig. S5).

For the short Gd ruler (2.1 nm) clearer modulations and narrower distance distributions are observed as the frequency offset is increased. Clearly visible modulations in the time domain are observed, at the largest PO offset of 900 MHz (see Fig. 5a), although simulations have indicated that some residual effects from the pseudo-secular term can be observed even at such PO offsets (Manukovsky et al., 2017). Interestingly, small artefacts are even observed for the longer Gd ruler (6.0 nm) in Fig. 3 where better fits to the expected Pake pattern are obtained at the larger 420 MHz frequency offset, and the related distance distribution has its peak at the expected 6.0 nm distance (see Fig. 4b).

We note that in all the DEER studies reported so far, the central $\left|-\frac{1}{2}\rangle \to \left|\frac{1}{2}\rangle \right.\right.$ transition has always been selected, either for the pump or the observer pulse. It had generally been assumed that there would otherwise be too big a sensitivity penalty, and the advantage of operating at high fields was achieving a higher degree of excitation of the central transition by either pump or observer pulses. This led to the view that it is necessary to choose a Gd spin label with a large ZFS when measuring short distances to reduce the effect of unwanted flip flops (Dalaloyan et al., 2015).

In this present work, we also investigated the DEER set-up where the pump and observer pulses are placed on either side of the central transition, thus avoiding any excitation of the central transition completely (see Fig. S6a). These DEER experiments, P ${}_{3}$ O and P ${}_{4}$ O, shown in Fig. 7a, now show time domain data with clear oscillations smoothly damped to the limit value (modulation depth), giving clear Pake patterns and narrow distance distributions that are strikingly similar to those obtained for structurally related nitroxide rulers with comparable spin–spin distances (Godt et al., 2006). This is evidence that when the central transition does not play a role in the Gd ${}^{3+}$ –Gd ${}^{3+}$ DEER measurements, the mixing of states has no major effect, as they do not share energy levels with those involved in the pump and observer transitions.

The ability to measure shorter distances with Gd-based spin labels accurately has implications for sensitivity. Gd ruler (2.1 nm) would be expected to have at least 10 times higher echo SNR, compared to Gd ruler (6.0 nm) just from the shorter time window required for the DEER measurement, based on relaxation measurements at the central transition. This increase in sensitivity is likely to be even larger for biological samples that are usually highly protonated and thus have significantly shorter phase memory times. The relative loss of sensitivity is therefore much less when shorter time windows become feasible, as shown with Gd ruler (2.1 nm) where an echo SNR of 8330, as well as a modulation depth of 2.1 %, was obtained after only 1 h and a half, even with a reduced 1 kHz SRR.

For observer measurements made away from the central transition the relative gain, at short distances, also becomes larger as relaxation times are expected to shorten due to transition-dependent phase relaxation (Raitsimring et al., 2014). In the measurements presented here, this is partially offset by the increased bandwidth required and the reduced power available from the EIK–isolator–switch combination at the band edges of the EIK amplifier. The available power as a function of frequency is shown in Fig. S1b. However, interestingly, and perhaps counter-intuitively, we see little degradation in SNR, when neither pump nor observer is placed at the central transition. Modulation depth, although reduced, is still reasonable, and thus we still obtain excellent overall sensitivity under this condition.

We therefore predict that Gd systems with smaller ZFS than used here (see Table S1) are to be preferred because not only is it easier to avoid the central transition, but we would also expect the amplitude of other transitions to increase (per unit bandwidth), which will further increase sensitivity. We would also predict that relaxation effects due to thermally assisted fluctuations in the ZFS will reduce (Raitsimring et al., 2014). This is the subject of further investigation.

It should also be noted, when pumping away from the central transition, at 40 µM molecular concentration, the required background correction to DEER traces is small relative to the experimental modulation depth. Indeed, the requirement for background correction is almost eliminated at short distances, as can be seen from the raw traces provided in Fig. S3b. This is particularly important for Gd ${}^{3+}$ –Gd ${}^{3+}$ DEER measurements where modulation depths are low, as small errors in background correction can make a significant contribution to uncertainties in the calculated distance distribution. The results can be compared to the larger corrections required at slightly higher spin label concentrations when pumping on the central transition (Dalaloyan et al., 2015).

There is also scope to further improve sensitivity, at W-band, by increasing both the shot repetition rate and averaging times and operating at lower temperatures if the central transition is not excited. Backshort positions in the sample holder (see Fig. S1a) were also optimised for cross-polar isolation rather than matching out the echo signal, which can make a difference of a factor of 2 in sensitivity. Other groups have demonstrated a significant sensitivity benefit from the use of broadband chirped pulses in DEER measurements on Gd ${}^{3+}$ systems (Bahrenberg et al., 2017; Doll et al., 2015). These methodologies particularly lend themselves to high-power, wideband spectrometers like HiPER and promise significant further gains. In contrast, we found both sensitivity and signal fidelity were significantly reduced at Q-band relative to W-band, for the case where both pump and observer are placed on one side of the central transition (due to cavity bandwidth limitations). Example data sets, measured using a high-power (150 W) commercial Q-band spectrometer with comparable sample volumes, are shown in Fig. S7 and Table S4 for Gd ruler (2.1 nm). For the case where both pump and observer are offset to one side of the central transition, resolution was only partially improved and sensitivity was reduced by a factor of 24 relative to W-band, using our defined sensitivity measure.

The sensitivity achieved at W-band suggests that it will be feasible to obtain high-quality spectra for Gd ${}^{3+}$ DEER measurements at sub- µM concentrations, even allowing for the shorter relaxation times commonly observed with spin-labelled biological samples. We have also observed promising results with Gd ${}^{3+}$ spin-labelled biological samples, which we will report in a future publication.

## 5 Conclusion

In the present work, we have investigated two Gd rulers, with Gd–Gd distances of 2.1 and 6.0 nm, using Gd ${}^{3+}$ complexes with a moderate ZFS of 1060 MHz. We have performed a variety of Gd ${}^{3+}$ –Gd ${}^{3+}$ DEER measurements with different offsets between pump and observer pulses, using a non-resonant induction mode cavity. This is a flexible wideband measurement set-up with relatively easy sample handling, where an excellent signal-to-noise ratio is observed.

We have shown, in agreement with previous experimental results, that larger PO offsets significantly reduce the artefacts observed for Gd–Gd distances below 3–4 nm, but they appear also to be of benefit in the case of larger distances, such as 6.0 nm.

More importantly we have shown significantly improved distance distributions at short distances by completely avoiding excitation of the central transition in the DEER experiment, $\left|-\frac{1}{2}\rangle \to \left|\frac{1}{2}\rangle \right.\right.$ , and mostly selecting $\left|-\frac{7}{2}\rangle \to \left|-\frac{5}{2}\rangle \right.\right.$ , $\left|-\frac{5}{2}\rangle \to \left|-\frac{3}{2}\rangle \right.\right.$ and $\left|-\frac{3}{2}\rangle \to \left|-\frac{1}{2}\rangle \right.\right.$ transitions. This still gives very high signal-to-noise ratio (per unit measurement time) while obtaining much improved fitting to expected Pake patterns. This is a strong motivation to select and/or develop Gd-based SLs with as small a ZFS as possible and measure using wideband spectrometers at moderately high magnetic fields, where the central transition narrows. The sensitivity is already high, but we envisage considerable scope for improvement.

## Supplement

10.5194/mr-1-301-2020-supplement The supplement related to this article is available online at: https://doi.org/10.5194/mr-1-301-2020-supplement.

## Data Availability

The research data underpinning this publication can be accessed at https://doi.org/10.17630/96ab76ee-38f4-468f-9ea8-e947f638261f (EL Mkami et al., 2020).

## References

[bib1.bib1] Bahrenberg T, Rosenski Y, Carmieli R, Zibzener K, Qi M, Frydman V, Godt A, Goldfarb D, Feintuch A (2017). Improved sensitivity for W-band Gd(III)-Gd(III) and nitroxide-nitroxide DEER measurements with shaped pulses. J Magn Reson.

[bib1.bib2] Clayton JA, Keller K, Qi M, Wegner J, Koch V, Hintz H, Godt A, Han S, Jeschke G, Sherwin MS, Yulikov M (2018). Quantitative analysis of zero-field splitting parameter distributions in Gd(iii) complexes. Phys Chem Chem Phys.

[bib1.bib3] Cohen MR, Frydman V, Milko P, Iron MA, Abdelkader EH, Lee MD, Swarbrick JD, Raitsimring A, Otting G, Graham B, Feintuch A, Goldfarb D (2016). Overcoming artificial broadening in Gd
${}^{3+}$
-Gd
${}^{3+}$
 distance distributions arising from dipolar pseudo-secular terms in DEER experiments. Phys Chem Chem Phys.

[bib1.bib4] Collauto A, Frydman V, Lee MD, Abdelkader EH, Feintuch A, Swarbrick JD, Graham B, Otting G, Goldfarb D (2016). RIDME distance measurements using Gd(iii) tags with a narrow central transition. Phys Chem Chem Phys.

[bib1.bib5] Cruickshank PA, Bolton DR, Robertson DA, Hunter RI, Wylde RJ, Smith GM (2009). A kilowatt pulsed 94GHz electron paramagnetic resonance spectrometer with high concentration sensitivity, high instantaneous bandwidth, and low dead time. Rev Sci Instrum.

[bib1.bib6] Dalaloyan A, Qi M, Ruthstein S, Vega S, Godt A, Feintuch A, Goldfarb D (2015). Gd(III)-Gd(III) EPR distance measurements – the range of accessible distances and the impact of zero field splitting. Phys Chem Chem Phys.

[bib1.bib7] Dalaloyan A, Martorana A, Barak Y, Gataulin D, Reuveny E, Howe A, Elbaum M, Albeck S, Unger T, Frydman V, Abdelkader EH, Otting G, Goldfarb D (2019). Tracking Conformational Changes in Calmodulin in vitro, in Cell Extract, and in Cells by Electron Paramagnetic Resonance Distance Measurements. Chem Phys Chem.

[bib1.bib8] Doll A, Qi M, Wili N, Pribitzer S, Godt A, Jeschke G (2015). Gd(III)-Gd(III) distance measurements with chirp pump pulses. J Magn Reson.

[bib1.bib9] EL Mkami H, Hunter RI, Cruickshank PAS, Taylor MJ, Lovett JE, Feintuch A, Qi M, Godt A, Smith GM (2020). University of St Andrews Research Portal.

[bib1.bib10] Feintuch A, Otting G, Goldfarb D (2015). Gd
${}^{3+}$
 Spin Labeling for Measuring Distances in Biomacromolecules: Why and How?. Methods Enzymol.

[bib1.bib11] Garbuio L, Zimmermann K, Häussinger D, Yulikov M (2015). Gd(III) complexes for electron-electron dipolar spectroscopy: Effects of deuteration, pH and zero field splitting. J Magn Reson.

[bib1.bib12] Godt A, Schulte M, Zimmermann H, Jeschke G (2006). How Flexible Are Poly(para-phenyleneethynylene)s?. Angew Chem Int Edit.

[bib1.bib13] Goldfarb D (2014). Gd
${}^{3+}$
 spin labeling for distance measurements by pulse EPR spectroscopy. Phys Chem Chem Phys.

[bib1.bib14] Gordon-Grossman M, Kaminker I, Gofman Y, Shai Y, Goldfarb D (2011). W-Band pulse EPR distance measurements in peptides using Gd
${}^{3+}$
dipicolinic acid derivatives as spin labels. Phys Chem Chem Phys.

[bib1.bib15] Jeschke G, Polyhach Y (2007). Distance measurements on spin-labelled biomacromolecules by pulsed electron paramagnetic resonance. Phys Chem Chem Phys.

[bib1.bib16] Jeschke G, Chechik V, Ionita P, Godt A, Zimmermann H, Banham J, Timmel CR, Hilger D, Jung H (2006). DeerAnalysis2006 – a comprehensive software package for analyzing pulsed ELDOR data. Appl Magn Reson.

[bib1.bib17] Kathirvelu V, Sato H, Eaton SS, Eaton GR (2009). Electron spin relaxation rates for semiquinones between 25 and 295 K in glass-forming solvents. J Magn Reson.

[bib1.bib18] Keller K, Mertens V, Qi M, Nalepa AI, Godt A, Savitsky A, Jeschke G, Yulikov M (2017). Computing distance distributions from dipolar evolution data with overtones: RIDME spectroscopy with Gd(iii)-based spin labels. Phys Chem Chem Phys.

[bib1.bib19] Manukovsky N, Feintuch A, Kuprov I, Goldfarb D (2017). Time domain simulation of Gd
${}^{3+}$
-Gd
${}^{3+}$
 distance measurements by EPR. J Chem Phys.

[bib1.bib20] Martorana A, Bellapadrona G, Feintuch A, Di Gregorio E, Aime S, Goldfarb D (2014). Probing Protein Conformation in Cells by EPR Distance Measurements using Gd
${}^{3+}$
 Spin Labeling. J Am Chem Soc.

[bib1.bib21] Meyer A, Schiemann O (2016). PELDOR and RIDME Measurements on a High-Spin Manganese(II) Bisnitroxide Model Complex. J Phys Chem A.

[bib1.bib22] Motion CL, Cassidy SL, Cruickshank PAS, Hunter RI, Bolton DR, El Mkami H, Van Doorslaer S, Lovett JE, Smith GM (2017). The use of composite pulses for improving DEER signal at 94 GHz. J Magn Reson.

[bib1.bib23] Pannier M, Veit S, Godt A, Jeschke G, Spiess HW (2000). Dead-Time Free Measurement of Dipole-Dipole Interactions between Electron Spins. J Magn Reson.

[bib1.bib24] Potapov A, Yagi H, Huber T, Jergic S, Dixon NE, Otting G, Goldfarb D (2010). Nanometer-scale distance measurements in proteins using Gd
${}^{3+}$
 spin labeling. J Am Chem Soc.

[bib1.bib25] Qi M, Groß A, Jeschke G, Godt A, Drescher M (2014). Gd(III)-PyMTA Label Is Suitable for In-Cell EPR. J Am Chem Soc.

[bib1.bib26] Qi M, Hülsmann M, Godt A (2016). Spacers for Geometrically Well-Defined Water-Soluble Molecular Rulers and Their Application. J Org Chem.

[bib1.bib27] Raitsimring A, Astashkin AV, Enemark JH, Kaminker I, Goldfarb D, Walter ED, Song Y, Meade TJ (2013). Optimization of pulsed DEER measurements for Gd-based labels: choice of operational frequencies, pulse durations and positions, and temperature. Appl Magn Reson.

[bib1.bib28] Raitsimring A, Dalaloyan A, Collauto A, Feintuch A, Meade T, Goldfarb D (2014). Zero field splitting fluctuations induced phase relaxation of Gd
${}^{3+}$
 in frozen solutions at cryogenic temperatures. J Magn Reson.

[bib1.bib29] Raitsimring AM, Astashkin AV, Poluektov OG, Caravan P (2005). High-field pulsed EPR and ENDOR of Gd
${}^{3+}$
 complexes in glassy solutions. Appl Magn Reson.

[bib1.bib30] Raitsimring AM, Gunanathan C, Potapov A, Efremenko I, Martin JML, Milstein D, Goldfarb D (2007). Gd
${}^{3+}$
 Complexes as Potential Spin Labels for High Field Pulsed EPR Distance Measurements. J Am Chem Soc.

[bib1.bib31] Razzaghi S, Qi M, Nalepa AI, Godt A, Jeschke G, Savitsky A, Yulikov M (2014). RIDME Spectroscopy with Gd(III) Centers. J Phys Chem Lett.

[bib1.bib32] Shah A, Roux A, Starck M, Mosely JA, Stevens M, Norman DG, Hunter RI, El Mkami H, Smith GM, Parker D, Lovett JE (2019). A Gadolinium Spin Label with Both a Narrow Central Transition and Short Tether for Use in Double Electron Electron Resonance Distance Measurements. Inorg Chem.

[bib1.bib33] Smith GM, Cruickshank PAS, Bolton DR, Robertson DA, Gilbert BC (2008). Electron Paramagnetic Resonance: Volume 21.

[bib1.bib34] Stoll S, Schweiger A (2006). EasySpin, a comprehensive software package for spectral simulation and analysis in EPR. J Magn Reson.

[bib1.bib35] Wojciechowski F, Groß A, Holder IT, Knörr L, Drescher M, Hartig JS (2015). Pulsed EPR spectroscopy distance measurements of DNA internally labelled with Gd
${}^{3+}$
-DOTA. Chem Commun.

[bib1.bib36] Yagi H, Banerjee D, Graham B, Huber T, Goldfarb D, Otting G (2011). Gadolinium tagging for high-precision measurements of 6nm distances in protein assemblies by EPR. J Am Chem Soc.

[bib1.bib37] Yang Y, Yang F, Li XY, Su XC, Goldfarb D (2019). In-Cell EPR Distance Measurements on Ubiquitin Labeled with a Rigid PyMTA-Gd(III) Tag. J Phys Chem B.

