# Supplementary material for: High-sensitivity Gd3+–Gd3+ EPR distance measurements that eliminate artefacts seen at short distances
Source: Magn Reson (Gott). 2020 Dec 9;1(2):301–13. doi: 10.5194/mr-1-301-2020 (PMC10500690; doi:10.5194/mr-1-301-2020)
Supplement: The supplement related to this article is available online at: https://doi.org/10.5194/mr-1-301-2020-supplement. [file mr-1-301-supplement.pdf]

Supplement of Magn. Reson., 1, 301–313, 2020
https://doi.org/10.5194/mr-1-301-2020-supplement
© Author(s) 2020. This work is distributed under
the Creative Commons Attribution 4.0 License.

Open Access
MAGNETIC RESONANCE

Supplement of

High-sensitivity Gd^{3+}–Gd^{3+} EPR distance measurements that eliminate artefacts seen at short distances

Hassane EL Mkami et al.

Correspondence to: Hassane EL Mkami (hem2@st-andrews.ac.uk) and Graham M. Smith (gms@st-andrews.ac.uk)

The copyright of individual parts of the supplement might differ from the CC BY 4.0 License.

System	D (mT)	FWHH (mT)-Q	FWHH (mT)-W	References
Gd-rulers with $[Gd^{III}(PyMTA)]$ as the spin label	37.4[1]	14.2	4.38	(this work)
[Gd-DOTA]	22.8	-	1.30	(Raitsimring et al., 2014)
[Gd-4-iodo-PyMTA]	40.6	-	4.00	(Dalaloyan et al., 2015)
[Gd-595]	22.9	-	1.23	(Collauto et al., 2016)
[Gd-538]	44.2	-	3.81	(Collauto et al., 2016)
[Gd-4MMDPA]	63.6	-	15.00	(Gordon-Grossman et al., 2011;Potapov et al., 2010)
[Gd-DO3A-BrPSPy]	51.2	-	6.40	(Yang et al., 2018)
[Gd.sTPATCN]	-	2.54	1.14	(Shah et al., 2019)
$[Gd\text{-}NO_3Pic]$	18.3	-	-	(Clayton et al., 2018)

Table S1: ZFS splitting parameter D and the full width half height estimated from the central lines of the ED-FS spectra recorded at T=10 K at Q and W-bands. It should be noted that FWHH depends on solvent and whether the complex is measured as such, or as attached to another molecule. The literature should be consulted for details.

[1] ZFS determined for Gd-ruler (2.1 nm) and Gd-ruler (6.0 nm) was found to be identical within the uncertainty intervals.

Compound	T_m^a(μs)	A_1(%)	T_m^b(μs)	A_2(%)	10% signal[1](μs)
Gd-ruler (6.0 nm)	4.6±0.3	45±2	13.7±0.1	55±2	18.2
Gd-ruler (2.1 nm)	9.1±0.3	78±2	13.7±0.1	22±2	20.6

Table S2: Echo decay parameters derived from the fit of the experimental data shown in Fig. S2a. The data were fitted to the sum of two exponential functions with fixed exponents of 1 and 2 and defined by the fitting function:

$$y=A_1.e^{-\left(\frac{t}{T_m^a}\right)^1}+A_2.e^{-\left(\frac{t}{T_m^b}\right)^2}$$

The fit was excellent (R^2 = 0.9999 and RMSE = 0.0020) and values for A_1, A_2, T_m^a and T_m^b for this fit are shown in the table. Examination of the residual shows white noise at the end of the trace and a small modulation related to the electron dipolar coupling at the beginning of the trace.

[1] Corresponds to the time, from the π/2 pulse, at which the echo has decayed to 10% of its initial value. These values are more representative of the echo decay.

Compound	T_1^a(μs)	A_1(%)	T_1^b(μs)	A_2(%)	T_1(μs)[1]
Gd-ruler (6.0 nm)	15.5±0.3	40±2	45.7±0.3	60±2	31.3±0.3
Gd-ruler (2.1 nm)	14.8±0.3	29±2	53.1±0.3	71±2	36.8±0.3

Table S3: Inversion recovery parameters derived from the fit of the experimental data shown in Fig. S2b, performed for both samples. The data were well fitted (R^2 = 0.9999 and RMSE = 0.0025) by a sum of two exponential functions defined by the fitting function.

$$y=A_1.[1-2*e^{-\left(\frac{t}{T_1^a}\right)}]+A_2[1-2*e^{-\left(\frac{t}{T_1^b}\right)}]$$

Values for A_1, A_2 and T_1^a and T_1^b for this fit are given in the table.

[1]T_1 values derived from a mono-exponential fitting function are also shown for comparison (with goodness of fit parameters R^2 = 0.9980 and RMSE = 0.0140).

**Offset[1] (MHz)**	**Obs[2] π(ns)**	**Pump[3] π(ns)**	**τ_2(μs)**	**Data points**	**SRR[4] (kHz)**	**λ (%)**	**Echo SNR**	**Time averaging**	**Number of averages[5]**	**Sensitivity measure[6]**
**<u>Gd-ruler (2.1 nm)</u>**										
240 (P_1O_1)	32	8	1.2	172	0.980	2.9	2000	0h28min (192 scans)	9600	4.50
240 (P_2O_2)	32	8	1.2	172	0.980	0.7	5000	24h20min (9980 scans)	499000	0.38

Table S4: Q-band experimental settings parameters used for DEER measurements on Gd-ruler (2.1 nm) shown in Fig. S7, and the associated modulation depths obtained by fitting the DEER data with DeerAnalysis (2019) (Jeschke et al., 2006). The interpulse delay τ_1 was set to 380 ns for all experiments. The sensitivity measure is defined as the echo SNR multiplied by the modulation depth divided by the square root of the total number of echo measurements. It should be noted that this does not take into account differences in excitation bandwidth of pump and observer pulses, which for Q-band measurements were partially limited by resonator bandwidth.

[1] Frequency separation between pump pulse set at position i (P_i) and observer pulse at position j (O_j).

[2,3] Observer and pump π pulse lengths. The observer π/2 pulse was always half the observer π pulse.

[4] SRR is the shot repetition rate.

[5] Number of averages calculated as: number of scans * number of shots per point.

[6] The sensitivity measure is calculated as $=\frac{\lambda * SNR(Echo)}{(\sqrt{total\ number\ of\ points\ measured})}$ where SNR (echo) is the ratio of the maximum echo height to the standard deviation of the noise. This is obtained by subtracting a smoothed fit from the data and then calculating the standard deviation from the resulting noise trace. The total number of points measured is the total number of averages per point multiplied by the number of points in a scan.

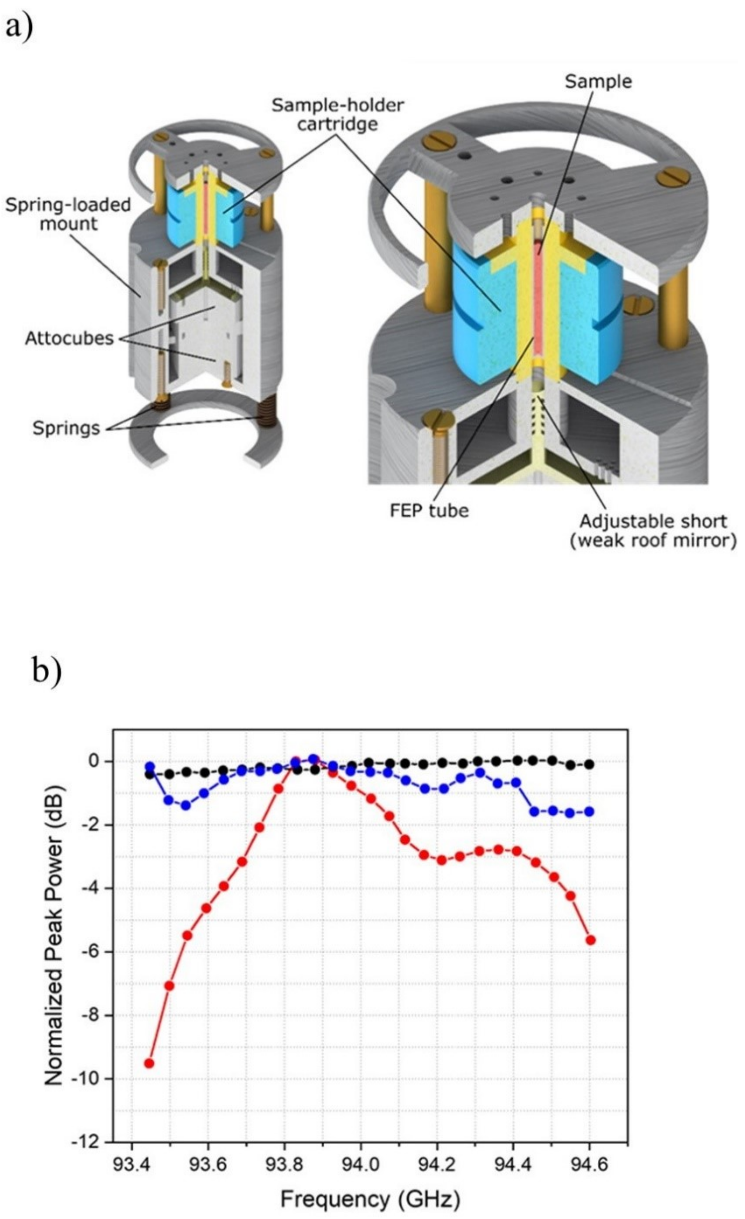

Fig. S1: a) CAD images of HiPER sample-holder showing the sample-holder cartridge mounted into the spring-loaded mount. b) Variation of the power level with respect to the frequency measured at different points in the transmit chain of HiPER. The measurements of the power output have been performed via couplers placed at the output of the multiplier (black), 1 W amplifier (blue) and 1 kW EIK amplifier (red). (Adapted from (Motion et al., 2017)).

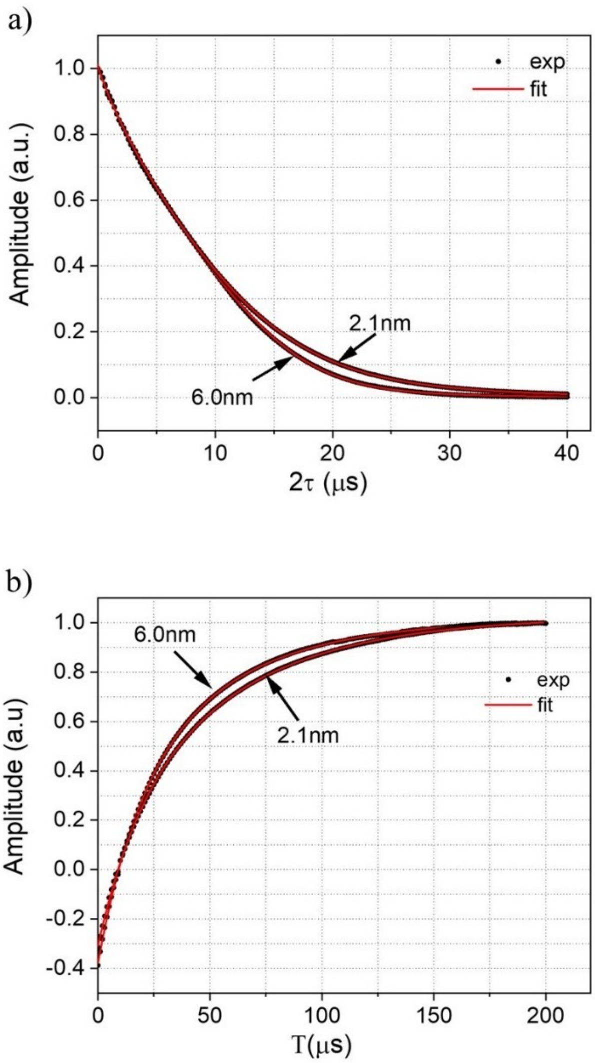

Fig. S2: Experimental and fitted a) echo decay and b) inversion recovery data recorded at 10 K at the maximum of the ED-FS spectra of Gd-ruler (2.1 nm) and Gd-ruler (6.0 nm). τ is the delay between the $\frac{\pi}{2}$ and π pulses in the echo decay pulse sequence and T is the delay between the inversion π pulse and $\frac{\pi}{2}$ - τ-π observer sequence. In a) the initial value of τ is set to 300 ns, and in b) τ is fixed to 300 ns and the initial value of T is set to 100 ns.

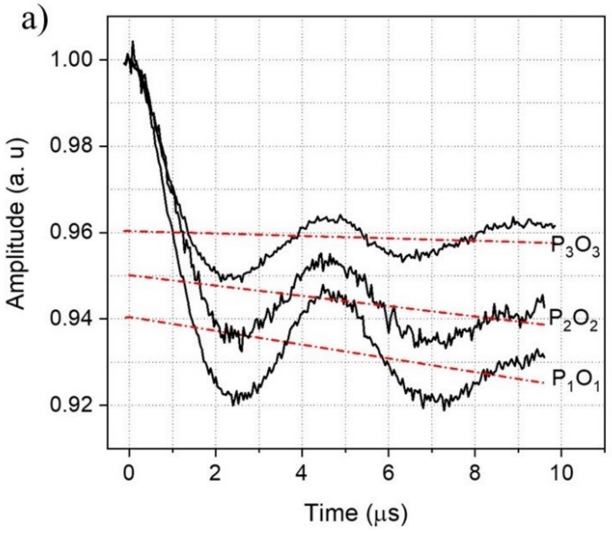

	Offsets	π_{obs}
**P_1O_1**	120 MHz	11 ns
**P_2O_2**	120 MHz	16 ns
**P_3O_3**	420 MHz	11 ns

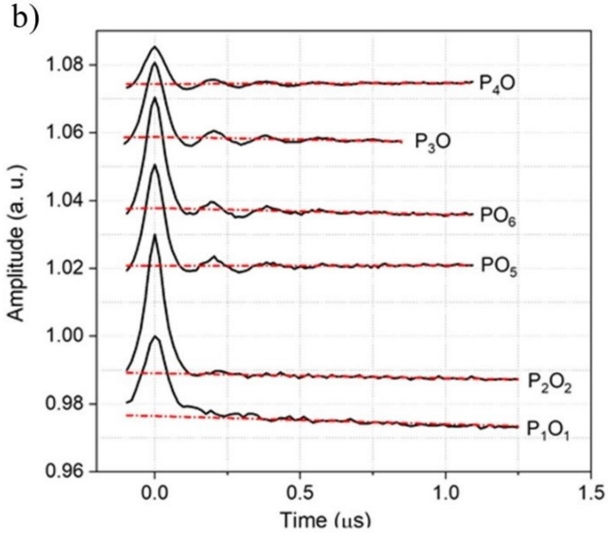

	Offsets	π_{obs}
**P_1O_1**	120 MHz	24 ns
**P_2O_2**	420 MHz	12 ns
**P_3O**	800 MHz	8 ns
**P_4O**	900 MHz	12 ns
**PO_5**	840 MHz	12 ns
**PO_6**	900 MHz	12 ns

Fig. S3: Primary DEER data of a) Gd-ruler (6.0 nm), and b) Gd-ruler (2.1 nm) recorded at different PO offsets. The background fitting used in DEER data analysis is shown in red. The frequency offsets corresponding to the different Pump/Observer positions are reported in tables next to each figure.

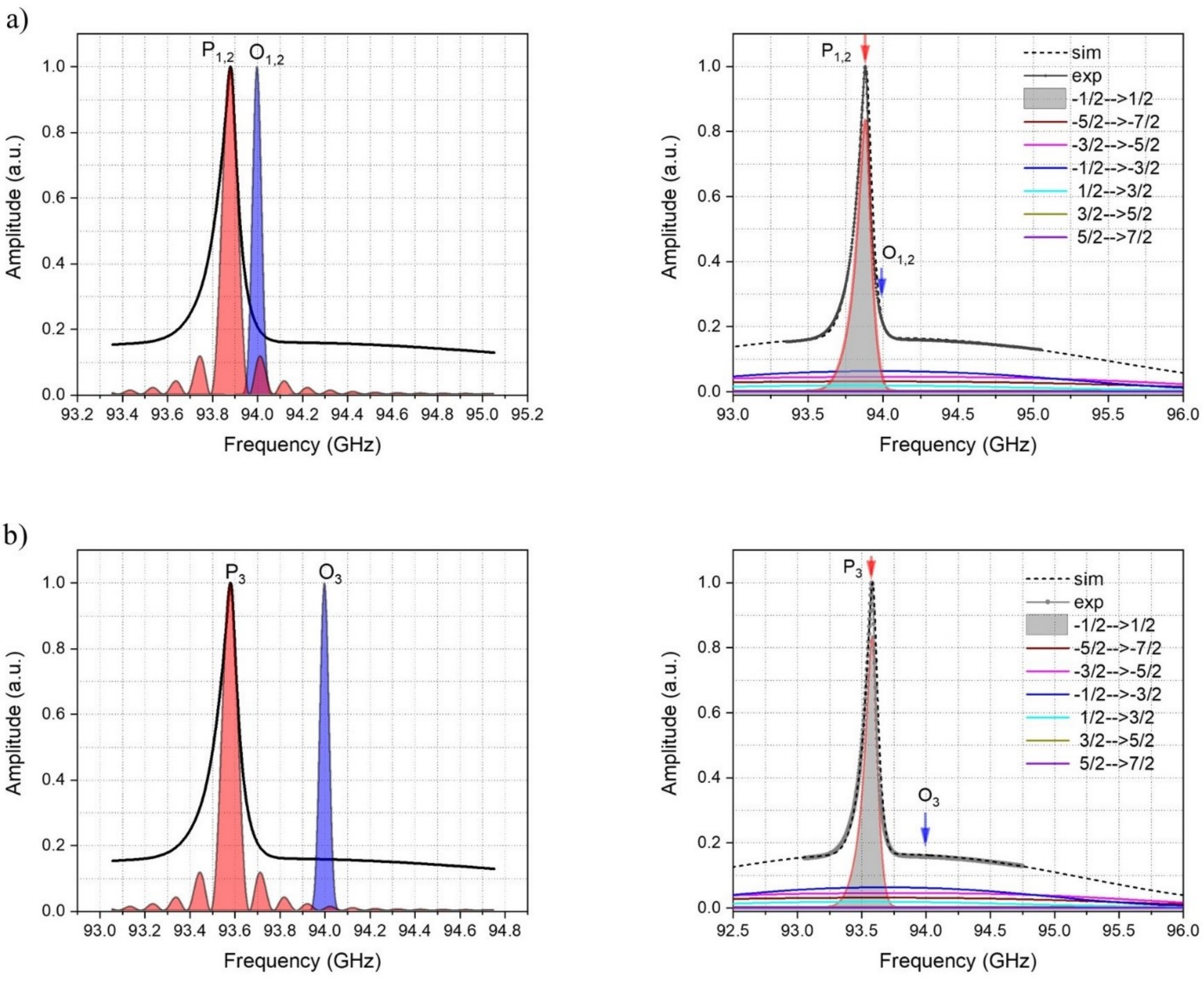

Fig. S4: ED-FS spectra of Gd-ruler (6.0 nm) sample with (left) simulated excitation profiles of the pump and observer pulses and (right) the associated field positions with respect to the highlighted central transition $\left|-\frac{1}{2}\right\rangle \rightarrow \left|\frac{1}{2}\right\rangle$. PO offsets: a) 120 MHz, b) 420 MHz. Note that pump frequencies are different although located at the central transition. The excitation plots in a) and b) are the same as those in the main text. They were added here for convenience.

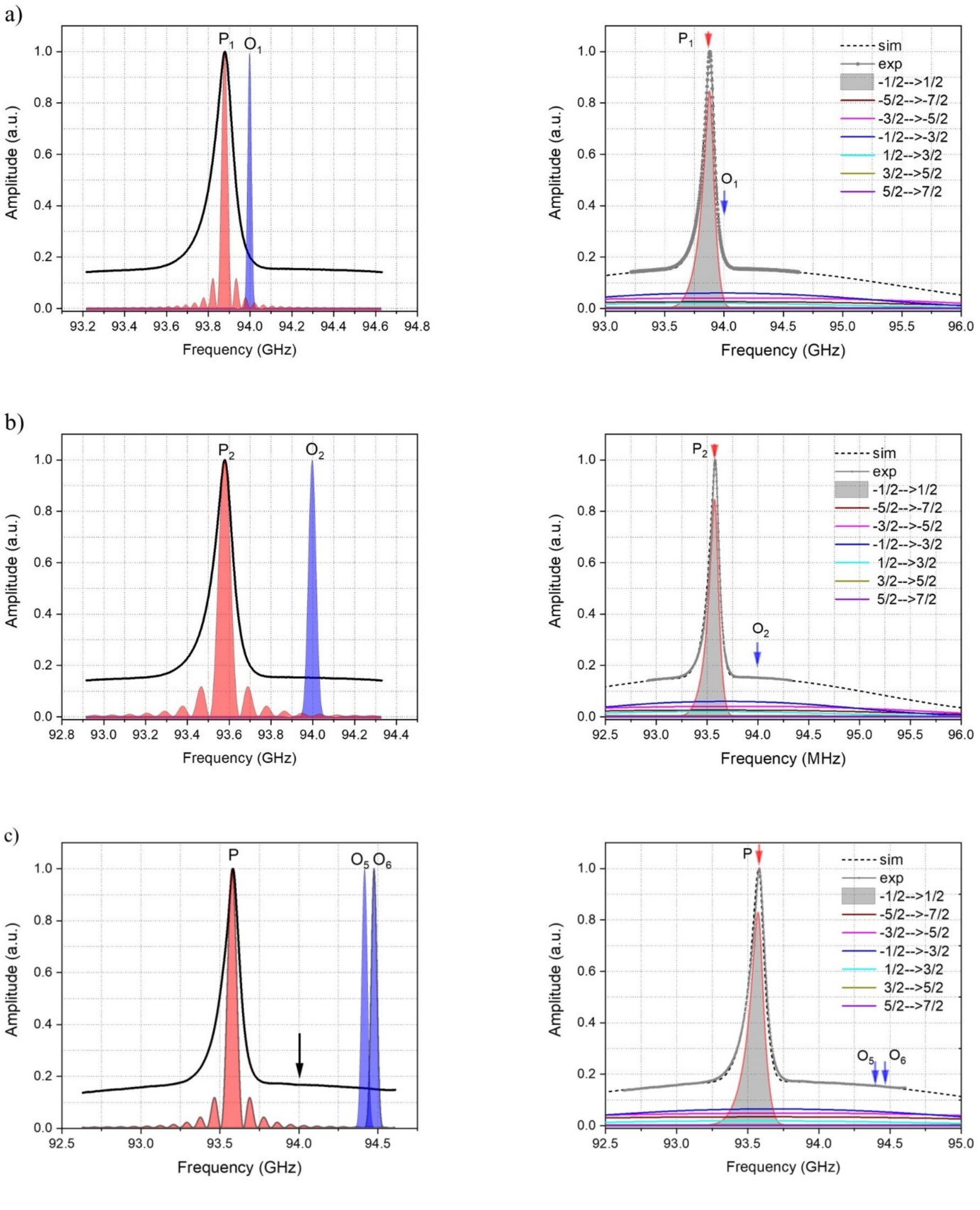
a)
P1
O1
Amplitude (a.u.)
Frequency (GHz)
sim
exp
-1/2-->1/2
-5/2-->-7/2
-3/2-->-5/2
-1/2-->-3/2
1/2-->3/2
3/2-->5/2
5/2-->7/2
b)
P2
O2
Amplitude (a.u.)
Frequency (GHz)
Frequency (MHz)
c)
P
O5
O6
Amplitude (a.u.)
Frequency (GHz)

Fig. S5: ED-FS spectra of Gd-ruler (2.1 nm) sample with (left) simulated excitation profiles of the pump and observer pulses and (right) the associated field positions with respect to the highlighted central transition $\left|-\frac{1}{2}\right\rangle \rightarrow \left|\frac{1}{2}\right\rangle$. PO offsets: a) 120 MHz, b) 420 MHz and c) 840 and 900 MHz. Note that pump frequencies are different although located at the central transition. The excitation plots are the same as those in the main text. They were added here for convenience. The black arrow indicates the position of 94 GHz, the nominal centre frequency of our W-band EIK amplifier, which has a bandwidth of just less than 1 GHz.

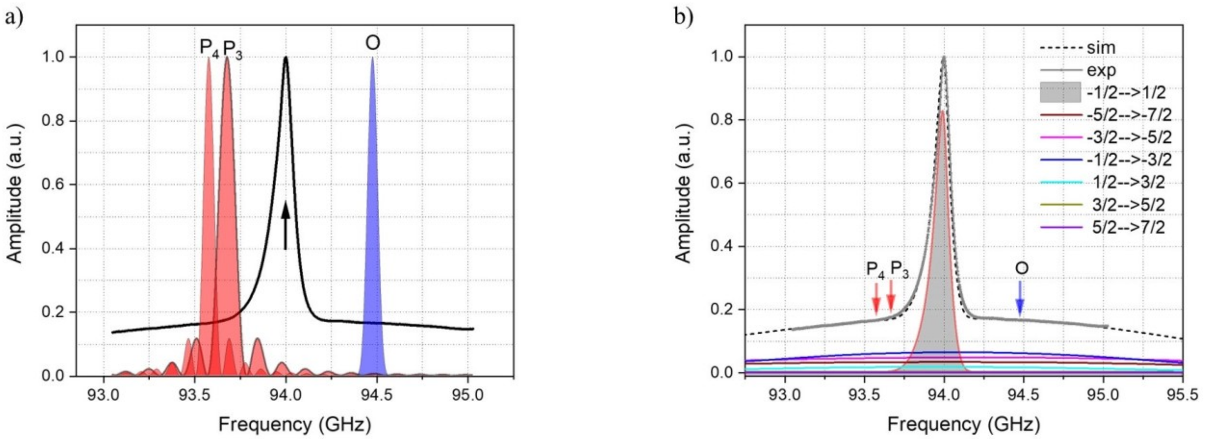

Fig. S6: ED-FS spectrum of Gd-ruler (2.1 nm) with a) simulated excitation profiles of the pump and observer pulses separated by 800 and 900 MHz and b) the pump and observer pulse field positions with respect to the highlighted central transition $\left|-\frac{1}{2}\right\rangle \rightarrow \left|\frac{1}{2}\right\rangle$. The excitation plots are the same as those in the main text. They were added here for convenience. The black arrow indicates the position of 94 GHz, the nominal centre frequency of our W-band EIK amplifier, which has a bandwidth of just less than 1 GHz.

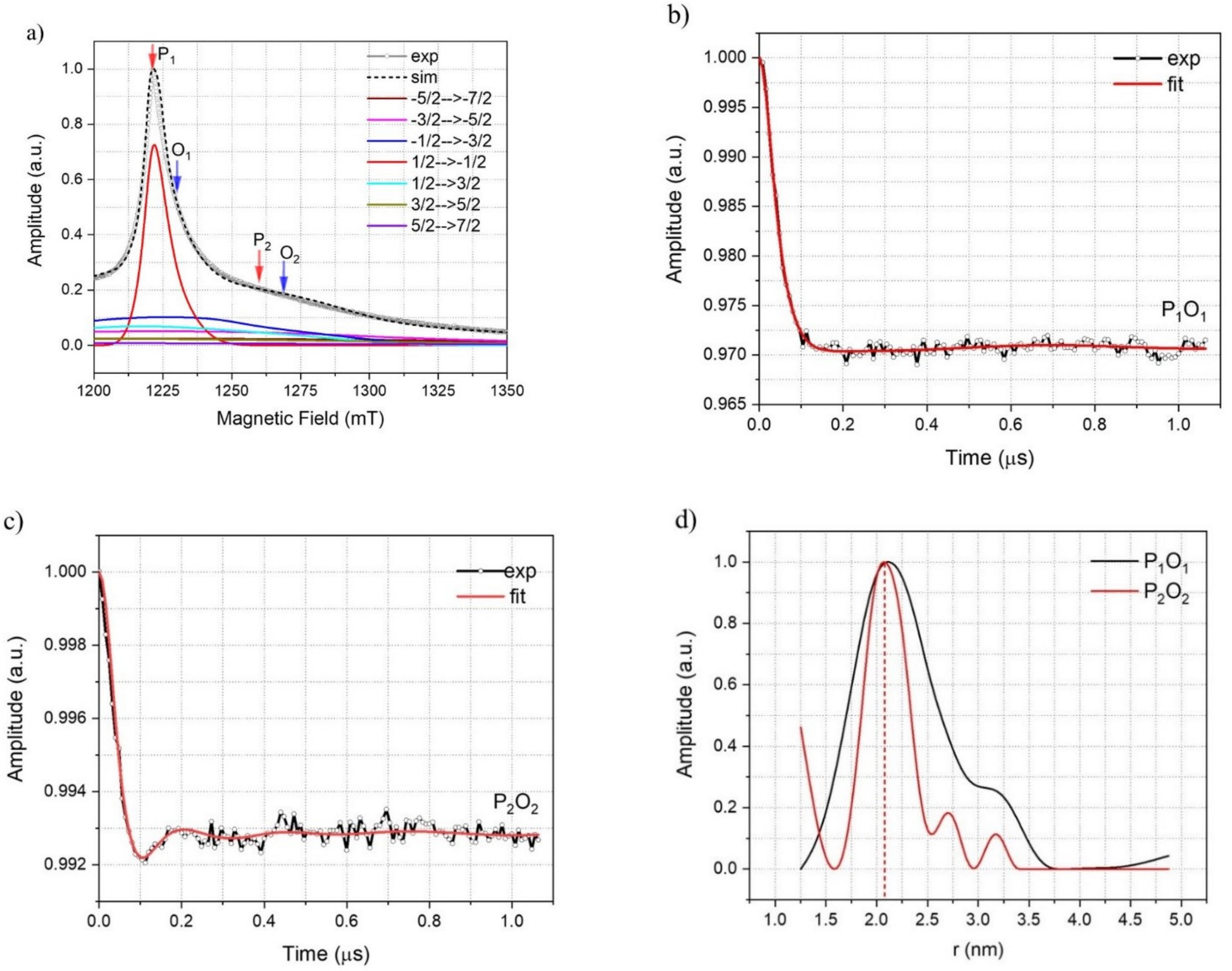

Fig. S7: a) Q-band ED-FS spectrum of Gd-ruler (2.1 nm) showing two different pump and observer position pairs, both with a PO offset of 240 MHz. The associated corrected background DEER data performed at P_1O_1 and P_2O_2 are shown in b) and c). Their corresponding distance distributions are shown in d). It should be noted that positioning both pump and observer on one side of the central transition was required by the limited bandwidth of the Q-band resonator. The Q-band data in Fig. S7c can be compared to the W-band data in Fig. 7a in the main paper, where the sensitivity was also 24 times larger.

References

Clayton, J. A., Keller, K., Qi, M., Wegner, J., Koch, V., Hintz, H., Godt, A., Han, S., Jeschke, G., Sherwin, M. S., and Yulikov, M.: Quantitative analysis of zero-field splitting parameter distributions in Gd(iii) complexes, Physical Chemistry Chemical Physics, 20, 10470-10492, https://doi.org/10.1039/C7CP08507A, 2018.

Collauto, A., Feintuch, A., Qi, M., Godt, A., Meade, T., and Goldfarb, D.: Gd(III) complexes as paramagnetic tags: Evaluation of the spin delocalization over the nuclei of the ligand, Journal of Magnetic Resonance, 263, 156-163, https://doi.org/10.1016/j.jmr.2015.12.025, 2016.

Dalaloyan, A., Qi, M., Ruthstein, S., Vega, S., Godt, A., Feintuch, A., and Goldfarb, D.: Gd(III)-Gd(III) EPR distance measurements--the range of accessible distances and the impact of zero field splitting, Phys Chem Chem Phys, 17, 18464-18476, https://doi.org/10.1039/c5cp02602d, 2015.

Gordon-Grossman, M., Kaminker, I., Gofman, Y., Shai, Y., and Goldfarb, D.: W-Band pulse EPR distance measurements in peptides using Gd(3+)-dipicolinic acid derivatives as spin labels, Phys Chem Chem Phys, 13, 10771-10780, https://doi.org/10.1039/c1cp00011j, 2011.

Jeschke, G., Chechik, V., Ionita, P., Godt, A., Zimmermann, H., Banham, J., Timmel, C. R., Hilger, D., and Jung, H.: DeerAnalysis2006—a comprehensive software package for analyzing pulsed ELDOR data, Applied Magnetic Resonance, 30, 473-498, https://doi.org/10.1007/BF03166213, 2006.

Motion, C. L., Cassidy, S. L., Cruickshank, P. A. S., Hunter, R. I., Bolton, D. R., El Mkami, H., Van Doorslaer, S., Lovett, J. E., and Smith, G. M.: The use of composite pulses for improving DEER signal at 94GHz, Journal of Magnetic Resonance, 278, 122-133, https://doi.org/10.1016/j.jmr.2017.03.018, 2017.

Potapov, A., Yagi, H., Huber, T., Jergic, S., Dixon, N. E., Otting, G., and Goldfarb, D.: Nanometer-scale distance measurements in proteins using Gd3+ spin labeling, J Am Chem Soc, 132, 9040-9048, https://doi.org/10.1021/ja1015662, 2010.

Raitsimring, A., Dalaloyan, A., Collauto, A., Feintuch, A., Meade, T., and Goldfarb, D.: Zero field splitting fluctuations induced phase relaxation of Gd3+ in frozen solutions at cryogenic temperatures, J Magn Reson, 248, 71-80, https://doi.org/10.1016/j.jmr.2014.09.012, 2014.

Shah, A., Roux, A., Starck, M., Mosely, J. A., Stevens, M., Norman, D. G., Hunter, R. I., El Mkami, H., Smith, G. M., Parker, D., and Lovett, J. E.: A Gadolinium Spin Label with Both a Narrow Central Transition and Short Tether for Use in Double Electron Electron Resonance Distance Measurements, Inorganic Chemistry, 58, 3015-3025, https://doi.org/10.1021/acs.inorgchem.8b02892, 2019.

Yang, Y., Yang, F., Gong, Y.-J., Bahrenberg, T., Feintuch, A., Su, X.-C., and Goldfarb, D.: High Sensitivity In-Cell EPR Distance Measurements on Proteins using an Optimized Gd(III) Spin Label, The Journal of Physical Chemistry Letters, 9, 6119-6123, https://doi.org/10.1021/acs.jpclett.8b02663, 2018.
